# Nutrient-dense snack bars from biofortified crops to enhance school children’s micronutrient intake in Tanzania

**DOI:** 10.1371/journal.pone.0346034

**Published:** 2026-04-01

**Authors:** Anselm P. Moshi, Ladislaus Kasankala, Analice Kamala, Nyabasi Makori, Dyness Kejo, Hoyce Mshida, Beatrice Bachwenkizi, Malimi Kitunda, Francis Millinga, Ai Zhao, Germana H. Leyna

**Affiliations:** 1 Tanzania Food and Nutrition Centre, Dar-es salaam, Tanzania; 2 Vanke School of Public Health, Tsinghua University, Beijing, China; 3 Department of Epidemiology & Biostatistics, Muhimbili University of Health and Allied Sciences, Dar es Salaam, Tanzania; Lusofona University of Humanities and Technologies: Universidade Lusofona de Humanidades e Tecnologias, PORTUGAL

## Abstract

Recent national surveys show that anaemia affects approximately 30–40% of school-aged children and up to 50% of adolescents in Tanzania, with prevalence exceeding 50% in some regions, underscoring the urgent need for nutrient-dense, school-appropriate food solutions. This study developed snack bars from biofortified crops and compared partially germinated and non-germinated formulations to improve the diets of school-aged children and adolescents. Germination significantly (P ≤ 0.05) increased carbohydrate, fat, fibre, and provitamin A content. Zinc content was slightly higher in the germinated bars, whereas iron content was slightly lower compared to the non-germinated formulation. Both formulations supplied 50–70% of the Recommended Dietary Allowances for carbohydrate, protein, fibre, iron and zinc iron aligning with Tanzania’s School Feeding Policy targets. Sensory testing showed higher acceptance of germinated ingredient’s snack bars for texture, colour, aroma and overall acceptability, while non-germinated bars were preferred for appearance and taste. These results demonstrate that both germinated and non-germinated biofortified snack bars can provide substantial amounts of essential nutrients in a child-friendly form. To our knowledge, this is the first study in Tanzania to develop germinated biofortified crop–based snack bars, offering a scalable, culturally acceptable approach to reducing micronutrient deficiencies among school-aged children.

## 1. Introduction

Micronutrient deficiencies are among the most common forms of malnutrition globally [1,2]. Over 2 billion people globally suffer from micronutrient deficiencies, with millions of children facing food insecurity due to climate crises, rising food prices, unhealthy food availability, undiversified diets, and inadequate child feeding practices [3]. Micronutrient deficiencies such as iron, vitamin A, vitamin B_12_, folate and zinc are particularly prevalent in low- and middle-income countries, Tanzania included [4,5]. The insufficient intake, absorption, or utilization of essential vitamins and minerals, lead to impaired immune function, cognitive dysfunction, and increased morbidity and mortality [6]. Iron deficiency anaemia remains a major global challenge, affecting about 42% of children worldwide [7]. Among adolescent girls, prevalence varies widely from around 26% in Europe and Central Asia to as high as 52% in West and Central Africa [8]. In East Africa, other micronutrient deficiencies are equally alarming: vitamin A deficiency ranges from 4% to 71%, while zinc deficiency affects between 3% and 76% of the population [8].

School-aged children (6–19 years) are particularly vulnerable due to their increased nutritional needs for growth and development [9]. In Tanzania, nearly 42% of children are at risk of zinc deficiency, which weakens immune function [10]. Anaemia among adolescents remains a significant public health concern in Tanzania. National estimates indicate that approximately one-third of adolescents are affected, with marked regional variation. Prevalence ranges from about 13% in Iringa to over 50% in the Coastal (Pwani) region. These pronounced geographic disparities reflect underlying differences in dietary diversity, infection burden, and regional food systems, highlighting the need for targeted, context-specific nutrition interventions [11–14].

When disaggregated by age, micronutrient deficiency prevalence exceeds 50% among adolescents aged 15–19 years and is consistently higher in rural than urban areas for both boys and girls [15]. These disparities are largely driven by poor dietary diversity, with over 70% of energy intake derived from staple foods low in essential micronutrients [11]. As a result, many children and adolescents experience stunting, anaemia, and impaired cognitive and physical development, undermining human capital and national productivity [16]. Tanzania’s dietary and demographic context reinforces the need for nutrient-dense interventions. Diets are dominated by maize, rice, and cassava, with limited intake of animal-source foods, fruits, and vegetables, contributing to widespread micronutrient inadequacies [15]. At the same time, Tanzania has a youthful population, with about 40% under 15 years, and steadily rising school enrolment. Between 2014/15 and 2020/21, net enrolment rose substantially across all education levels in Tanzania, increasing by 9.5 percentage points in pre-primary, 8.4 points in primary, and 14.3 points in secondary education nationwide, with especially strong gains in rural areas and the largest increases observed in urban secondary enrolment [17]. Together, these patterns highlight the strong potential reach of school-based nutrition programs. In this context, fortified snack bars delivering 50–70% of selected RDAs represent a strategically relevant and highly scalable option within the national school feeding system.

In response, Tanzania’s government has implemented strategies such as dietary diversification, micronutrient supplementation, food fortification, and crop biofortification to combat malnutrition and promote long-term socio-economic development [12,14]. Despite the government’s efforts and commitments, nutrient deficiencies remain widespread in Tanzania. One contributing factor to these persistent challenges may be the implementation approach, as most nutrition interventions have primarily targeted children under five and pregnant or lactating women. As a result, school-aged children (6–9 years) and adolescents (10–18 years) have been largely overlooked [8]. To address this gap, the Government initiated the school feeding program to ensure that these age groups are not left behind. However, for the program to succeed, the school food environment must be strengthened, particularly in the areas of food procurement, nutritional quality, safety, and consistent adequacy of meals provided.

While biofortification holds potential, smallholder farmers have not scaled up sufficiently to meet national needs [18]. Meeting the nutritional needs of school-aged children and adolescents is critical for improving health and strengthening the country’s human capital. Tanzania has prioritized tackling malnutrition and micronutrient deficiencies through national policies such as the National Nutrition Strategy (2011/12–2015/16) and the Tanzania Food and Nutrition Policy (1992). These policies aim to enhance nutrition-sensitive interventions, including the promotion of biofortified foods and other food-based solutions.

Recent studies show that food-based interventions effectively improve micronutrient status among school-aged children. Fortified snacks including iron- and zinc-fortified bars [19], high-energy fortified biscuits in school feeding programs [20], and calcium-fortified nutritious biscuits [21] are well accepted and enhance nutrient intake. Broader reviews and policy analyses confirm that fortified snack products are practical, child-friendly vehicles easily integrated into school feeding initiatives [22]. Compared with porridge or beverages, fortified snack bars offer key advantages: they are ready-to-eat, shelf-stable, and require no preparation, reducing logistical and contamination challenges [23]. Their fixed portion size ensures consistent micronutrient dosing, unlike porridges subject to dilution variability [24], while high acceptability improves uptake and program coverage [21,22].

Utilizing locally available biofortified crops such as yellow maize, Jesca/TAR-6 beans, and orange-fleshed sweet potatoes offers an innovative, cost-effective way to develop nutrient-dense foods for vulnerable school-aged children and adolescents. Since snacks are a preferred and convenient food choice for this age group, they provide an effective vehicle for delivering essential micronutrients that can improve health, academic performance, and long-term productivity. Therefore, this study aimed to develop and evaluate nutrient-dense snack bars formulated from locally available biofortified crops, and to assess their nutritional composition and sensory acceptability among school-aged children. This approach was intended to address existing gaps in adolescent nutrition interventions in Tanzania and to contribute to improved health outcomes and long-term socio-economic development.

## 2. Materials and methods

### 2.1. Source of raw material

The raw materials used for the formulation of the High Iron and Zinc Snack Bar (HIZSB) included biofortified yellow maize, Jesca/TARI 6 biofortified beans, green gram, sesame, and pumpkin seeds. All raw materials were procured from contracted smallholder farmers through a small- to medium-scale nutritious food processing enterprise, BIVAC Tanzania Limited, which operates under the incubation of the Tanzania Engineering and Manufacturing Design Organization (TEMDO) in Arusha, Tanzania. Ripe bananas (Malindi cultivar) and pineapples were purchased from local wet markets in Arusha city, while ice sugar was sourced from a local supermarket.

### 2.2. Preparation of material

All raw materials, including cereals, legumes, sesame, and pumpkin seeds, were sorted, washed with tap water, and dried to approximately 12% moisture content. The cereals and legumes were then divided into two portions: one portion underwent partial germination, while the other remained non-germinated.

#### 2.2.1. Partial germination of cereals and legumes.

Cereals (maize) and legumes (beans, green gram) were uniformly spread on muslin cloth (0.5–0.8 cm thickness). Water was gently sprinkled to moisten the seeds, and samples were placed in a dark environment to promote germination. Maize and green gram germinated for 48 h, whereas beans required 60 h. Germinated ingredients were dehydrated using a mechanical dehydrator to reduce moisture content to ~12%, preventing over-fermentation or spoilage.

#### 2.2.2. Roasting and size reduction.

Cereals and legumes were roasted in a custom-built roaster at 100–130 °C for 15–20 min, cooled to room temperature, and milled using a small hammer mill. The resulting flour was sieved through a 0.5 mm sieve. Sesame and pumpkin seeds were pulverized in a high-speed blender to achieve a fine consistency. Pineapples were peeled, chopped, and blended into a puree, then strained through cheesecloth to produce juice. Ripe bananas were peeled, blended, and used directly without straining to preserve texture and nutrient content.

### 2.3. Raw material and finished products composition analysis

#### 2.3.1. Proximate analysis.

Moisture content was determined by drying samples at 105 °C for 3 h. Ash content was measured after heating samples at 550 °C for 5 h [25]. Protein content of each sample was determined by a standardised Kjeldahl method AOAC (2005) [26]. Crude fibre content was determined using a standardized procedure [25]. Fat content was determined using the Soxhlet extraction method (AOAC, 2005) [26]. Carbohydrate content was calculated by difference:


% Carbohydrate = 100−(Moisture+Ash+Protein+fat+ fibre)

#### 2.3.2. Determination of zinc and iron.

Iron and zinc concentrations were quantified using Microwave Plasma-Atomic Emission Spectroscopy (MP-AES). Duplicates of 2 g samples were ashed at 550 °C for 4 h. Ash was eluted with nitric and hydrochloric acid, diluted to 100 mL, and 50 mL aliquots were analysed using an Agilent 4200 MP-AES [27].

#### 2.3.3. Determination of vitamin A in provitamin A maize.

Vitamin A in provitamin A maize was determined following a method adapted from [28]. Briefly, 2 g of homogenized sample was extracted with acetone, saponified with 80% KOH, and repeatedly extracted with hexane. The combined hexane extract was evaporated under nitrogen, reconstituted in methanol, and analysed using HPLC (LC-20A, SHIMADZU).

### 2.4. Product formulation

#### 2.4.1. The theoretical formulation.

A theoretical formulation for HIZSB was developed using food composition data (Table 1) with the aim of meeting 50–70% of the RDA for iron, zinc, vitamin A, and folate. Linear programming identified optimal nutrient combinations, resulting in five prototype formulations. Daily portion size for school-aged children and adolescents was set at 200 g.

**Table 1:** Theoretical prototype formulation based on food composition tables and %RDA for age groups.

		% RDA for age groups 6–9 and 10–18 years		
Prototype 1	Nutrient values	Children (6 –8) years	Boys (10 –18) years	Girls (10 –18) years
Energy (kcal)	730.62	44	26	31
Carbohydrate (g)	90.64	39	21	26
(Protein (g))	31.99	49	30	32
(Fat (g))	20.22	44	31	37
(Fibre (g))	29.06	94	63	71
(Iron (mg))	13.53	68	50	52
(Zinc (mg))	6.75	84	52	56
(Vitamin A (µg RAE))	142.05	17	14	16
(Folate (µg DFE))	350.35	72	51	55
**Cost (TZS)**	**458.00**			
**Prototype 2**				
Energy (kcal)	675.88	41	24	26
Carbohydrate (g)	90.44	39	21	26
Protein (g)	37.38	58	35	37
Fat (g)	11.3	25	17	21
Fibre (g)	31.45	101	68	77
Iron (mg)	13.14	66	49	51
Zinc (mg)	7.25	91	56	60
(Vitamin A (µg RAE))	120.03	14	12	13
(Folate (µg DFE))	468.77	97	68	73
**Cost (TZS)**	**464.40**			
**Prototype 3**				
Energy (kcal)	668.38	40	23	28
Carbohydrate (g)	85.25	37	20	24
Protein (g)	40.86	63	39	40
Fat (g)	10.84	24	17	20
Fibre (g)	33.19	107	72	81
Iron (mg)	13.39	67	50	52
Zinc (mg)	7.45	93	57	62
(Vitamin A (µg RAE))	95.94	11	9	11
(Folate (µg DFE))	543.76	112	79	85
**Cost (TZS)**	**490.80**			
**Prototype 4**				
Energy (kcal)	665.13	40	23	28
Carbohydrate (g)	83.89	36	19	24
Protein (g)	40.97	63	39	41
Fat (g)	10.79	23	17	20
Fibre (g)	34.29	111	75	84
Iron (mg)	14.08	70	52	54
Zinc (mg)	7.62	95	59	64
(Vitamin A (µg RAE))	89.64	10	9	10
(Folate (µg DFE))	518.06	107	75	81
**Cost (TZS)**	**505.20**			
**Prototype 5**				
Energy (kcal)	669.56	40	24	28
Carbohydrate (g)	85.85	37	20	25
Protein (g)	40.65	63	38	40
Fat (g)	10.88	24	17	20
Fibre (g)	32.83	106	71	80
Iron (mg)	13.21	66	49	51
Zinc (mg)	7.4	93	57	62
(Vitamin A (µg RAE))	98.7	12	10	11
(Folate (µg DFE))	545.9	113	79	85
**Cost (TZS)**	**486.00**			

Key: The % RDA was calculated based on the Tanzania Mainland Food-Based Dietary Guidelines, Ministry of Health (2023) [29].

NB: Cost provided are Ex-factory (ingredients and all other operational costs at factory).

#### 2.4.2. Formulation using actual nutrient composition.

The nutrient content of raw materials was analysed, and linear programming was applied to determine actual proportions for five prototype formulations (Table 2). Prototypes 2 and 4, as well as 3 and 5, had similar nutrient profiles; prototypes 4 and 5 were therefore eliminated. Remaining prototypes (1 –3) were further optimized to balance nutrient content and sensory quality for the target population.

**Table 2:** Prototype formulation based on actual nutrient composition of ingredients.

	None Germinated					Germinated				
Ingredient	P1	P2	P3	P4	P5	P1	P2	P3	P4	P5
Maize (%)	45.45	36.36	27.27	25.45	28.18	45.45	36.36	27.27	25.45	28.18
Bean (%)	15.91	24.09	30	36.36	28.18	15.91	24.09	30	36.36	28.18
Green gram (%)	15.91	24.09	27.27	22.73	28.18	15.91	24.09	27.27	22.73	28.18
Sesame (%)	9.09	1.82	1.82	1.82	1.82	9.09	1.82	1.82	1.82	1.82
P. seed (%)	4.55	4.55	4.55	4.55	4.55	4.55	4.55	4.55	4.55	4.55
Ice Sugar (%)	9.09	9.09	9.09	9.09	9.09	9.09	9.09	9.09	9.09	9.09

P1-P5 = Prototypes.

#### 2.4.3. Development of product prototype.

Ingredients were mixed according to predetermined ratios. Prototype 3 was divided into two portions to compare germinated (P3_G) and non-germinated cereals and legumes (P3_NG), while Prototype 2 remained non-germinated (P2_NG) (Table 3). Dough was mixed at 1000 rpm for 14 min, hand-kneaded, shaped with moulds, and baked at 150 °C for 55 min. Phase one sensory evaluations guided modifications in taste, colour, shape, and texture, including reduction of particle size from 0.8 mm to 0.5 mm and addition of banana pulp (12.6%) and pineapple juice (16%) to improve flavour and aroma. Therefore, protype P2 (NG) was dropped and Prototype P3 (GM and NG) were modified to produce the final product.

**Table 3:** Split plot design to determine the effect of Germination of Cereals and Legumes.

	Biofortified Yellow Maize (%)	Biofortified Bean (%)	Green gram (%)	Sesame (%)	P. seed (%)	Icing Sugar (%)
Prototype 3_NG	27.27	30	27.27	1.82	4.55	9.09
Prototype 3_GM	27.27	30	27.27	1.82	4.55	9.09
Prototype 2_NG	36.36	24.09	24.09	1.82	4.55	9.09

Whereby, “NG” refers to non-germinated cereals and legumes, while “G” refers to germinated cereals and legumes.

**Fig 1:**
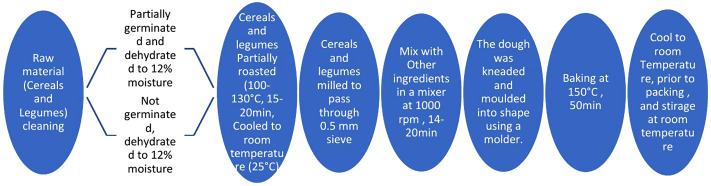
Process flow and Conditions.

The complete process flow and processing conditions are presented in Fig 1.

### 2.5. Sensory evaluation

Sensory evaluation was conducted three times during prototype development by 12 trained panellists (school children 15–19 years old), twice at BIVAC TEMDO Arusha and once at TFNC, Dar-es-Salaam. Snack bars were assessed for texture, colour, taste, aroma, and overall acceptability using a nine-point hedonic scale [24]. Samples were served in coded, randomized order. The recruitments of panellists were done from 20^th^ to 23^rd^ April 2024 and 2^nd^ to 5^th^ May 2024 for Arusha and Dar-es salaam participants respectively.

### 2.6. Ethical considerations

The study was approved by the National Institute for Medical Research (approval SZEC-2439/R.A/V.1/188). Informed consent was obtained from all participants in accordance with the Declaration of Helsinki (World Medical Association, (2013). All participants in the sensory evaluation study were fully informed about the purpose, procedures, and voluntary nature of their involvement. Informed consent was obtained in written form prior to participation. No minors were included in the sensory evaluation, so parental or guardian consent was not required. All consent was obtained directly from the adult participants, and no waivers of consent were sought or granted by the ethics committee.

### 2.7. Statistical analysis

Compositional analysis results are presented as mean ± SD from triplicate determinations. Sensory evaluation data were analysed using ANOVA, and Tukey’s Studentized Range Test was used for post-hoc pairwise comparisons where significant differences (p ≤ 0.05) were detected.

## 3. Results and discussion

**Fig 2:**
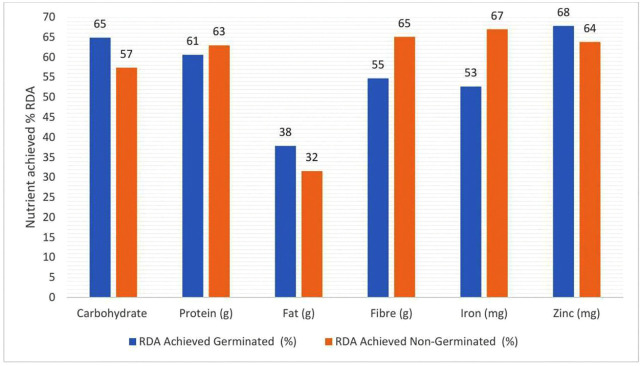
Percentage of required daily allowance for school children and adolescents achieved in snack bar.

A nutrient-dense snack bar has been developed using linear programming techniques, optimized to incorporate locally available, biofortified crops rich in essential micronutrients. The formulation ensures that a daily intake of 200 grams provides approximately 50–70% of the recommended dietary allowance (RDA) for key nutrients (Carbohydrate, Protein, Fibre, Iron and Zinc) in school-aged children and adolescents (Fig 2). The intervention was designed in collaboration with a local food processing small and medium-sized enterprise (SME), facilitating a clear pathway from research to commercial production. The snack bars are intended for integration into the national school feeding program, enabling widespread distribution.

This food-based strategy targets the reduction of micronutrient deficiencies among children aged 6–19 years, with a projected scale-up potential to reach over 25 million beneficiaries across Tanzania. Beyond addressing undernutrition, the product is positioned as a healthier alternative to commonly consumed, nutrient-poor snacks such as high-sugar and deep-fried items currently prevalent in school environments.

### 3.1. Composition of raw material

The raw materials used in the formulation of the nutrient-dense snack bar, aimed at addressing micronutrient deficiencies among school-aged children, were selected based on their micronutrient content, particularly iron, zinc, provitamin A, and fibre. Table 4 presents the nutritional composition of the key ingredients, including biofortified maize flour, biofortified beans flour, sesame seeds, pumpkin seeds, and green gram flour, in terms of moisture content, crude protein, crude fibre, fat, iron, zinc, provitamin A, and folate.

**Table 4:** Composition of raw material used in the Snack.

Raw Material	Maize Flour		Beans Flour		Green Gram Flour		High grade sesame	High Pumpkin seeds
	NG	G	NG	G	NG	G		
Moisture content (%)	10.6 ± 0.7	11.6 ± 0.3	13.4 ± 0.9	13.4 ± 0.9	10.2 ± 0.1	9.6 ± 0.4	3.3 ± 0.0	6.2 ± 0.1
Ash (%)	2.4 ± 0.3	3.1 ± 0.5	2.9 ± 0.3	3.1 ± 0.4	3.7 ± 0.1	3.9 ± 0.2	11.6 ± 0.4	4.1 ± 0.2
Carbohydrate (g/100g)	70.2 ± 0.0	67.3 ± 0.8	52.85 ± 1.9	51.6 ± 2.9	53.1 ± 0.4	52.4.4 ± 0.6	30.5 ± 0.0	12.3 ± 0.0
Crude Protein (g/100g)	8.5.0 ± 0.2	9.5 ± 0.1	23.4 ± 2.1	24.2 ± 2.2	24.3 ± 0.0	25.1 ± 0.1	14.9 ± 0.1	25.6 ± 0.2
Crude Fibre (g/100g)	3.8 ± 0.2	4.0 ± 1.0	5.3 ± 0.4	5.7 ± 0.4	4.8 ± 0.0	5.0 ± 0.3	4.4 ± 0.0	2.6 ± 0.1
Fat (g/100g)	4.5 ± 0.3	4.5 ± 0.5	2.15 ± 0.8	2.0 ± 0.8	3.9 ± 0.5	4.0 ± 0.5	35.3 ± 0.2	49.2 ± 0.1
Iron (mg/100g)	3.4 ± 2.7	1.7 ± 0.5	9.0 ± 1.9	7.3 ± 0.2	3.7 ± 1.9	3.9 ± 0.3	5.4 ± 0.3	8.7 ± 0.1
Zinc (mg/100g)	4.9 ± 0.4	2.6 ± 1.3	5.2 ± 0.2	4.9 ± 0.6	5.1 ± 0.3	4.9 ± 0.5	2.3 ± 0.9	2.10 ± 1.1
Provitamin A (µg/100g)	7.9 ± 1.1	7.4 ± 1.8						
Folate (µg/100g)					0.1 ± 0.8	0.1 ± 0.8		

NG=None Germinated, G= Germinated.

#### 3.1.1. Effect of germination on nutritional composition.

Germination produced distinct, crop-specific changes (Table 4). Protein increased consistently in maize (8.5 → 9.5 g/100 g), beans (23.4 → 24.2 g/100 g), and green gram (24.3 → 25.1 g/100 g), while crude fibre rose slightly (+0.2–0.4 g/100 g) (Table 4). Carbohydrates declined in legumes (beans −0.8 g/100 g, green gram −2.0 g/100 g) but were stable in maize (75.0 → 75.2 g/100 g) (Table 4), reflecting carbohydrate mobilization during sprouting. Fat changed minimally (≈ −0.15 to +0.10 g/100 g), while ash content increased (+0.2–0.7%) (Table 4) suggesting improved mineral concentration. Mineral changes were more mixed: iron decreased sharply in maize (3.4 → 1.7 mg/100 g) and beans (9.0 → 7.3 mg/100 g), though green gram showed a slight increase (3.7 → 3.9 mg/100 g); zinc decreased modestly in all crops (−0.2 to −2.3 mg/100 g) (Table 4). For vitamins, provitamin A in maize declined slightly (7.9 → 7.4 µg/100 g), and folate remained low (~0.1 µg/100 g) (Table 4) with no significant change. (Sesame and pumpkin seeds were not germinated and thus serve as nutrient-dense, stable ingredients in the formulation).

These findings align with recent evidence showing that germination induces favourable biochemical changes in cereals and legumes, enhancing nutritional quality in ways that support snack-bar fortification. For example, germination has been shown to increase protein solubility and in vitro protein digestibility in legumes by reducing antinutritional factors and promoting proteolysis of storage proteins [24,30,31,32]. Similarly, sprouting has been demonstrated to reduce phytic-acid and other chelators, thereby improving the bioavailability [30] and potential absorption of minerals such as iron and zinc even when total mineral content declines [26,27]. A recent germinated-quinoa study also found that germination enhanced mineral concentrations and improved the nutritional and functional properties of protein isolates, underscoring the value of germinated grains for nutrient-dense food formulations [31]. These compositional and bioavailability improvements support our rationale of combining germinated flours with nutrient-rich seeds in snack bar formulations aimed at improving protein, fibre, and mineral nutrition among school-aged children and adolescents.

#### 3.1.2. Effect of Germination on specific nutrients.

**Carbohydrates.** Germination had contrasting effects on cereals and legumes. In maize, total carbohydrate content increased marginally (75.2 vs. 74.6 g/100 g; Table 4), reflecting limited starch-to-sugar conversion during the 48-h sprouting period. Similar modest increases have been linked to activation of amylolytic enzymes, which hydrolyse starch into soluble sugars [33,34]. By contrast, legumes showed slight declines (22.7 → 21.9 g/100 g in beans; 63.4 → 61.4 g/100 g in green gram), consistent with starch mobilisation to support embryo growth [35].

**Protein.** Protein content increased modestly in maize (8.5 → 9.5 g/100 g) and more substantially in legumes (beans and green gram; Table 4). This aligns with previous reports that protease activity during germination breaks down storage proteins into soluble, more digestible forms, often enhancing limiting amino acids such as lysine [19,20,36]. Such improvements suggest better protein quality for human consumption [37].

**Fat.** Lipid content remained largely unchanged in maize, beans, and green gram, confirming that germination does not markedly alter fat concentration. However, shifts in fatty acid profiles toward higher unsaturation have been reported elsewhere [38], which may improve fat quality even in the absence of large compositional changes.

**Fibre.** Crude fibre showed small but notable increases, particularly in green gram (4.8 → 5.0g/100 g). This may reflect cell wall loosening and greater solubility of fibre fractions, as also reported in other legumes [39]. Increased fibre contributes positively to gut health and satiety.

**Minerals.** Absolute mineral concentrations, including iron and zinc, decreased slightly after germination (Table 4). However, this reduction is often offset by improved bioavailability due to phytate hydrolysis and reduction of other antinutrients [22,27]. In biofortified TAR6 beans, higher baseline iron and zinc levels may further counterbalance such small declines.

**Vitamins.** Germination enhanced B-vitamin content, particularly folate, which rose markedly in green gram (90 → 143 µg/100 g). This is consistent with prior work showing increased enzymatic synthesis and release of bound vitamins during sprouting [35]. Such gains are nutritionally relevant given widespread folate inadequacy in school-aged children.

Overall, germination induced nutrient shifts of modest magnitude in maize but more pronounced changes in legumes, especially increased protein quality, fibre, and B-vitamins. Although slight reductions in iron, zinc, and provitamin A were observed, improved mineral bioavailability and the inclusion of biofortified beans help maintain the nutritional integrity of the formulations. These compositional improvements underscore the potential of germinated legumes and biofortified beans to enhance the dietary quality of school snacks, aligning with efforts to address common micronutrient deficiencies in children.

#### 3.1.3. Sesame and pumpkin seeds.

Sesame (Sesamum indicum) and pumpkin (Cucurbita pepo) seeds are nutrient-dense ingredients that significantly enhance the nutritional profile of fortified snack formulations. Sesame seeds are rich in protein (14.9 g/100 g), fat (35.3 g/100 g), iron (53.5 mg/100 g), and zinc (22.8 mg/100 g), while pumpkin seeds provide higher fat content (49.2 g/100 g) and iron (86.9 mg/100 g), with comparable zinc levels (21.0 mg/100 g) (Table 4).

Both seeds contain substantial amounts of unsaturated fatty acids, which support brain development, immune function, and the absorption of fat-soluble vitamins such as provitamin A. The high iron and zinc content contributes to haemoglobin synthesis, immune competence, and growth, addressing common micronutrient deficiencies in children [28,29]. Incorporation of sesame and pumpkin seeds into snack bars helps reductions in mineral content observed during germination of cereals and legumes, while improving mineral bioavailability. When combined with biofortified maize, TAR6 beans, and green gram, these seeds contribute to a nutrient-dense, micronutrient-rich snack tailored to the dietary needs of school-aged children.

### 3.2. Nutrient composition of the finished product

Table 5 presents the nutrient composition of snack bars prepared from germinated and non-germinated ingredients. Significant differences (p < 0.05) were observed, with the germinated snack bar showing higher carbohydrates, fats, Energy (kcal), zinc, provitamin A, moisture, and ash, but slightly lower protein, fibre, and iron.

**Table 5:** Nutritional composition of the snack bar.

Nutrients	Germinated Ingredients (Mean ± SDV)	Non-Germinated ingredients (Mean ± SDV)	P-Value
Energy (kcal)/100g	363.1 ± 0.13	328.5 ± 0.21	0.23
Carbohydrates (%)	42.2 ± 0.14	37.3 ± 0.58	0.000
Total Crude Proteins (%)	15.2 ± 0.36	15.8 ± 0.36	0.239
Total Crude Fat (%)	13.3 ± 0.18	11.1 ± 0.16	0.000
Total Saturated Fatty Acid (SFA)	1.6 ± 0. 31	1.31 ± 0. 26	0.0032
Total Monounsaturated Fatty Acid (MUFA)	3.1 ± 0. 25	2.56 ± 0. 18	0.320
Total Polyunsaturated Fatty Acid (PUFA)	7.6 ± 0. 43	6.32 ± 0. 51	0.045
Fibre (%)	6.9 ± 0.03	8.1 ± 0.21	0.000
Ash content (%)	10.5 ± 0.08	9.3 ± 0.25	0.002
Moisture content (%)	9.4 ± 0.01	8.3 ± 0.07	0.125
Iron (mg/100g)	4.0 ± 0.01	5.0 ± 1.36	0.185
Zinc (mg/100g)	3.7 ± 0.76	3.5 ± 0.23	0.503
Provitamin A (µg/100g)	0.86 ± 0.01	0.0 ± 0.00	0.000
Folate (µg/100g)	0.0 ± 0.00	0.0 ± 0.00	–

From nutrient composition derived in this study (Table 5) and applying standard Atwater factors (fat = 9 kcal/g; protein = 4 kcal/g; carbohydrate = 4 kcal/g; fibre = 2 kcal/g) [35,40,41,42], the 200 g ration provides 726 kcal for the germinated variant and 657 kcal for the non-germinated variant. Thus, germination improved calories content of the snack bars. When assessed against the Estimated Energy Requirements (EER) for children and adolescents aged 6–19 years [43], these snack bars contribute approximately 44% of daily energy needs for children aged 6–9 years (1661 kcal/day), 31% of daily needs for older girls aged 10–18 years (2387 kcal/day), and 26% for older boys aged 10–18 years (2846 kcal/day) [23]. Considering the nutrients, of interest (especially, iron and zinc) to address micronutrient deficiencies among school-aged children, their contributions align with the product’s design objective to deliver 50–70%. The portion size (200g/day) is delivered as several small bars (~20 g each) consumed gradually during the school day, ensuring feasibility and acceptability across age groups.

Germination slightly increased the caloric content of the snack bars by breaking down complex starches into more digestible sugars and partially solubilizing cell-wall polysaccharides, enhancing carbohydrate accessibility [35,44,45]. It may also modestly improve lipid and protein digestibility [46], which, combined with standard Atwater factors (fat = 9 kcal/g; protein = 4 kcal/g; carbohydrate = 4 kcal/g; fibre = 2 kcal/g) as recommended by FAO and Merrill & Watt [47,48,41,42], results in the higher calculated energy of the germinated bar (726 kcal/200 g) compared to the non-germinated variant (657 kcal/200 g).

#### 3.2.1. Proximate composition.

The germinated snack bar had increased carbohydrates (42.2% vs. 37.3%) and fats (13.26% vs. 11.07%), Energy (kcal) 726Vs. 657 reflecting starch breakdown into simpler sugars and the contribution of sesame and pumpkin seeds rich in unsaturated fatty acids [49,24]. Protein was slightly lower in the germinated bar (15.17% vs. 15.75%) due to enzymatic breakdown during germination [38] while fibre decreased modestly (6.84% vs. 8.14%), likely from partial hydrolysis. Ash content increased (10.45% vs. 9.31%), indicating improved mineral content and bioavailability due to reduced antinutrients [21,50]. Moisture content was slightly higher in the germinated bar (9.42% vs. 8.25%), consistent with water uptake during germination. Overall, both bars provide a balanced proximate composition suitable for supporting energy and growth in school-aged children.

#### 3.2.2. Micronutrients.

Iron was slightly lower in the germinated bar (3.95 vs. 5.03 mg/100 g), while zinc increased (3.73 vs. 3.51 mg/100 g), reflecting improved mineral bioavailability despite minor losses in total content [51]. Provitamin A was present only in the germinated bar (0.855 µg/100 g), derived from biofortified yellow maize, supporting vision, immunity, and skin health [52]. Folate was negligible in both bars, likely due to thermal processing; however, this highlights an opportunity for further fortification or complementary foods rich in folate, such as leafy greens or fruits [53,54].

Germination improved energy, healthy fats, and key micronutrients (zinc, provitamin A), enhancing the nutritional value of the snack bar. Both bars remain valuable sources of protein, fibre, and minerals for children, while the observed reduction in iron and absence of folate suggest areas for further formulation improvement or dietary pairing to optimize adolescent nutrition.

### 3.3. Percentage of recommended daily allowance contribution for school children and adolescents

The contribution of germinated and non-germinated snack bars to the RDA for **school-aged** children and adolescents (6–19 years) is summarized in Fig 2. Both bars provide substantial portions of key nutrients, highlighting their potential to address dietary gaps in low- and middle-income settings.

The germinated snack bar delivers 42.2% carbohydrates, covering 64.9% of the RDA, while the non-germinated bar provides 37.3%, meeting 57.4% of the RDA (Fig 2). These contributions are higher than previously reported for cereal-legume snacks [55]. Protein intake from the bars ranges between 60% and 63% of the RDA (Fig 2), supporting growth and development in school-aged children [56,57]. Fats are supplied at 37.9% of the RDA by the germinated bar and 31.6% by the non-germinated bar (Fig 2) largely derived from unsaturated fatty acids in sesame and pumpkin seeds, which are important for cognitive function [45]. Fibre content is significant, contributing 54.7% (germinated) and 65.1% (non-germinated) of the daily requirement, reflecting the high fibre content of the base ingredients [55].

For micronutrients, the snack bars provide substantial contributions to iron and zinc. Iron is supplied at 52.7% of the RDA by the germinated bar and 67.0% by the non-germinated bar. Germinated products are known to have reduced phytate and therefore the bioavailable iron is presumed to be higher in G than in NG foods [58,59]. Zinc intake ranges from 63.8% (non-germinated) to 67.9% (germinated), supporting growth and immune function. Moreover, with reduced phytate Zinc is presumed to be more available in the germinated in the germinated formulation and [54]. Provitamin A is present only in the germinated bar (0.855 µg/100 g), contributing to vision and immune health [52]. Folate is negligible in both bars due to thermal processing, identifying an opportunity for further fortification or combination with folate-rich foods to address deficiencies [54].

The macronutrient contributions observed in this study demonstrate that both the germinated and non-germinated snack bars deliver nutrient levels comparable to, and in some cases greater than, those reported in recent cereal–legume product development studies. The germinated bar provides 64.9% of the carbohydrate RDA and the non-germinated 57.4%, values higher than those reported for comparable grain-legume snacks formulated for school-aged children [60,61]. Protein contributions (60–63% of RDA) align closely with findings from [56,57,62] who similarly noted enhanced protein density in composite snacks containing legumes and oilseeds. Fat contributions (31.6–37.9% of RDA), largely from sesame and pumpkin seeds, mirror the lipid profiles described by Nsabimana et al. [63] who highlighted the cognitive benefits of unsaturated fatty acids in seed-based school snacks. Fibre content (54.7–65.1% of RDA) is consistent with the high fibre levels reported by [35] in germinated cereal–legume blends and reflects the intrinsic fibre density of beans and whole grains.

Micronutrient delivery from the snack bars was substantial, with iron contributions ranging from 52.7% to 67.0% of the RDA. This aligns with recent studies showing that germination and sprouting of legumes and cereals significantly reduce phytate content, thereby improving iron bio-accessibility and density in composite foods [63]. Similarly, zinc contributions (63.8–67.9% of RDA) are consistent with findings that germination enhances zinc bioavailability in legume-based foods [63]. The low folate content observed reflects the thermal sensitivity of folate, which is commonly degraded during processing [64]. The presence of provitamin A in the germinated variant is in line with modest carotenoid retention reported in germinated cereals [65]. Collectively, these data demonstrate that the snack bars effectively deliver key micronutrients and are consistent with contemporary evidence supporting germinated cereal–legume formulations as a strategy to improve iron and zinc intake in school-aged children.

Overall, both snack bars provide 50–70% of the RDA for essential nutrients, indicating their potential to reduce micronutrient deficiencies among school-aged children. When integrated into school-based nutrition programs and combined with complementary foods, these bars can significantly enhance dietary quality and support balanced growth and development.

### 3.4. Sensory characteristic of the finished products

**Fig 3:**
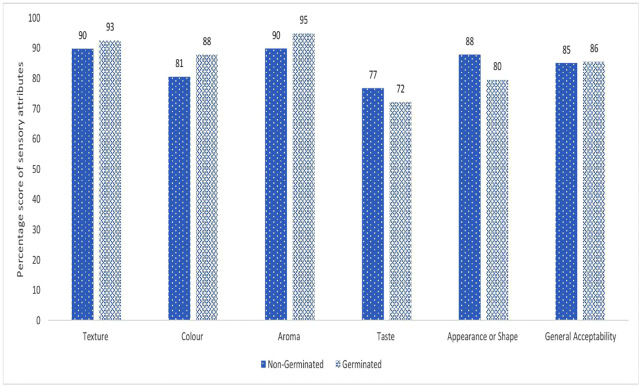
Sensory evaluation of the final germinated vz non germinated.

Several snack bar prototypes were developed using predetermined ratios of biofortified yellow maize, TAR6 beans, green gram, sesame, and pumpkin seeds, with variations in pre-treatment. Sensory evaluation of the final snack bars processed from germinated and non-germinated ingredients revealed distinct preferences across different attributes (Fig 3).

Fig 3 shows that germination improved texture (93% vs. 90%) and appearance (88% vs. 80%), colour (88 vs. 81), likely due to enzymatic modifications that enhance tenderness and visual appeal [66]. The germinated bar also scored higher for aroma (95vs.90) and slightly higher for general acceptability (86% vs. 85%), suggesting a smoother and cleaner taste profile resulting from reduced bitter compounds during germination [66]. The improved aroma can be attributed to the fact that germination can improve the aroma of cereal/legume-based foods by altering the volatile-compound profile, for example, in germinated seeds some undesirable “beany” volatiles decrease while more pleasant floral or nutty aroma compounds are produced [67].

Conversely, the non-germinated bar received slightly higher preference scores for taste (77% vs. 72%) and appearance (88% vs. 80%). This suggests that germination may introduce subtle changes in taste and visual characteristics that are less familiar to some consumers [15,68].

Overall, the sensory evaluation highlights a trade-off: germination enhances texture, appearance, and aftertaste, while slightly reducing aroma and taste. For school-aged children, texture and aftertaste may be more critical for acceptability than aroma, suggesting that the germinated snack bar is likely to be preferred by the target audience despite minor changes in flavour profile. Optimization of germination parameters or flavour enhancement strategies could further improve overall sensory acceptability.

## 4. Conclusion

In this study iron-zinc-rich snack bars were developed from germinated and non-germinated cereals and legumes to address micronutrient deficiencies among school-aged children in Tanzania and similar low- and middle-income settings. The snack bars provide balanced macronutrients (carbohydrates, protein, fats, and fibre) and key micronutrients, including iron, zinc, and provitamin A. The formulations deliver approximately 50–70% of the recommended daily allowance for most nutrients for children and adolescents aged 6–19 years, with carbohydrates contributing 57.4–64.9%, protein 61–63%, fibre 54.7–65.1%, iron 52.7–67.0%, and zinc 63.8–67.9% of daily requirements. Overall, both formulations demonstrate strong potential as nutrient-dense school snacks, although further optimization is required to maximize nutritional impact in resource-limited contexts. The snack bar formulated from partially germinated cereals and legumes demonstrated comparable overall nutritional suitability to the non-germinated formulation. The partially germinated snack bar showed comparable nutritional suitability to the non-germinated version. Although germination may enhance micronutrient bioavailability by reducing antinutrients, this was not directly measured and should therefore be considered a plausible, literature-supported benefit rather than a confirmed outcome of this study. Following efficacy and acceptability studies, the snack bars will be integrated into school routines through a coordinated, school-based distribution model, with trained small- and medium-scale enterprises producing and supplying the products under the supervision of the Tanzania Food and Nutrition Centre and municipal councils, ensuring consistent access, effective monitoring, and sustained uptake among school children and adolescents.

### Highlights

• Nutrient dense snack bar is formulated from bio-fortified locally available crops
• Snack bar provides 50–70% RDA of iron and zinc for children school-aged and adolescents
• Germinating cereals and legumes significantly enhanced the product’s nutritional profile
